# Erythemal UV radiation across Nigeria: where do we stand?

**DOI:** 10.1016/j.heliyon.2022.e10158

**Published:** 2022-08-11

**Authors:** Timothy C. Egbuim, Nnaemeka D. Onyeuwaoma, Bonaventure I. Okere, Mercy H. Ezenwugo, Augustina O. Chukwudi, Godspower O. Uhiene, Ngozi D. Ugwuozor, Baba I. Shaibu, Emeka A. Ugboma, Daniel R.E. Ewim

**Affiliations:** a Planetary Science Division, Centre for Basic Space Science, University of Nigeria, Nigeria; b Institute of Geophysics, Atmospheric Physics Department, University of Warsaw, Poland; c Department of Mechanical Engineering, Durban University of Technology, South Africa

**Keywords:** Erythemal, WHO, Latitude, Ultraviolet, Radiation, Public health

## Abstract

The deleterious effects of solar ultraviolet (UV) radiation on humans are of public health concern which demands constant global monitoring and intervention. This study analysed the latitudinal variations in mean daily and multi-year erythemal ultraviolet radiation (EUVR) across 7 states in Nigeria using satellite data from the Total Ozone Mapping Spectrophotometer (TOMS) and Ozone Mapping Instrument (OMI). The latitudes studied were 13° N, 8° N, 6° N and 4° N respectively, using 37 years of data obtained from phases: 1979–1988, 1989–1998, 1999–2008 and 2009–2020. The data obtained were statistically analysed using Ms Excel and MATLAB. The results of this study show that the mean daily EUVR at the selected latitudes ranges from 32.97 to 390 (mW/m^2^). The results show that Borno State located at latitude 13° N had the highest EUVR, while Rivers State recorded the least EUVR at latitude 4° N. Comparative analysis of all the locations studied indicates latitudinal and longitudinal variations because the eastern axis recorded higher values than its western counterpart on the same latitude. Box and whisker plots in this study summarized the latitudinal variance in the mean multi-year EUVR in each phase. Box and whisker plots from 2009–2020 showed that there was a drastic reduction in mean multi-year EUVR in this phase unlike in the other phases. The findings of this study when compared to the UV index show that the 37 years mean EUVR obtained across Nigeria ranged from 8–10 which is on the “very high” category. This study recommends the implementation of WHO suggestions in preventing the biological effects of solar UV radiation. Furthermore, the Nigerian government should curb ignorance among its citizens by heightening public awareness of the effects of EUVR.

## 1 Introduction

The biological effects of solar ultraviolet (UV) radiation are of public concern and according to Igbawua et al. (2013), even though solar UV radiation is responsible for the synthesis of vitamin D, excessive exposure to it causes sunburn (erythema), eye problems (such as cataracts, pterygium), skin ageing, skin cancer and immune depression. The effect of overexposure to solar UV radiation on humans has in recent years gained attention worldwide and requires more scientific intervention. The biological effects of solar UV radiation extend beyond our eco and aquatic systems, which affect both marine and freshwater habitats from essential biomass producers (phytoplankton) to consumers (zooplankton) in the food web (Hader et al., 2007).

According to Corrêa (2015), Solar UV radiation is generally divided into UV-A (315–400 nm), UV-B (280–315 nm) and UV-C (100–280 nm). The UV-C radiation does not reach the troposphere and the Earth's surface due to atmospheric absorption by oxygen and ozone (Kerr and Fioletov, 2008). The UV-B and UV-A reaching the earth's surface cause most biological damage in humans; at varying degrees. Park et al. (2015) reported that due to the varying effects of solar UV radiation, erythemal ultraviolet radiation (EUVR) was introduced to monitor and quantify the biological damages. EUVR is evaluated as the spectral UV irradiance weighted with the standard erythema action spectrum curve adopted by the Commission Internationale de l’Éclairage (CIE) in 1987; hence, the relative contribution of the irradiance at each wavelength to the induction of erythema on the human skin (McKinlay and Diffey, 1987; Fountoulakis et al., 2020; Webb et al., 2011). The minimal erythemal dose (MED) is the minimum amount of exposure to ultraviolet radiation that causes erythema of the skin (Gill and Kalia, 2015). Erythema occurs 24 h after exposure if the dose of erythemal irradiance has exceeded 1 unit of MED (Bilbao and de Migue, 2020), which depends on skin type and correlates with Fitzpatrick skin type classification. Among the light-skinned individuals, it ranges from 200 to 500 J/m^2^ and for the brown to dark-skinned individuals, MED ranges from 600 to 1000 J/m^2^ (Fitzpatrick, 1988; Gill and Kalia, 2015). The efficiency of producing erythemal increases by up to 5 orders of magnitude in the 280–350 nm wavelength range (Igbawua et al., 2013).

The MED even though widely used as a measure of EUVR is affected by various variables. These variables were accommodated by the CIE through the Standard Erythemal Dose (SED), the SED is a standardized measure of erythemogenic UV radiation (CIE, 2019). 1 SED is equivalent to an erythemal effective radiant exposure of 100 J/m^2^ and is independent of skin type (Salvadori et al., 2019). Naturally, the brown or black skin type is more resistant to instant sunburn or other acute effects of overexposure to solar UV radiation due to the dark skin pigment melanin (Badosa, 2005). This information has misled many, especially in Africa, making them vulnerable to the risks associated with excess exposure to solar UV radiation. The calculation of EUVR has been instrumental in the estimation of the UV index. According to (El-Nobi, 2018), the UV index expresses the erythemal power of the sun in W/m^2^ or mW/m^2^.

(1) $$UVI=40\times EUVR\;\left(\frac{W}{m^{2}}\right)=0.04\times EUVR\;\left(mW/m^{2}\right)$$

In 2002, the World Health Organization (WHO, 2002) adopted a global standardized public health measure for reporting UV irradiance (see Table 1), hence the need to carefully ascertain the level of EUVR exposures.

**Table 1:** The UV index categories (sources: Downs et al., 2016; WHO, 2002).

UVI	Category	Colour code	EUVR (mW/m^2^)
0–2	Low	Green	0 to 75
3–5	Medium	Yellow	75 to 150
6–7	High	Orange	150 to 200
8–10	Very High	Red	200 to 275
11+	Extreme	Purple	275+

Globally, the study of spatial distribution and variations of EUVR has attracted attention in countries such as Egypt (El-Nobi, 2018), Peninsular Malaysia (Shah et al., 2011; Tan et al., 2018), Slovakia (Pribullová and Chmelík, 2008), Thailand (Janjai et al., 2010), China (Liu et al., 2017), Italy (Fountoulakis et al., 2020), Spain (Utrillas et al., 2013) and Korea (Park et al., 2015). The study aims to analyse the latitudinal variations in EUVR across Nigeria. Therefore, in this research, we investigated the latitudinal variation of EUV Dose Rate (noon-time) over Nigeria between 1979 and 2020 using data from Total Ozone Mapping Spectrophotometer Nimbus-7 (TOMSN7: 1979–1993), TOMS Earth-Probe (TOMSEP: 1996–2005) and Ozone Monitoring Instrument (OMI: 2006–2020). The results of this research will help policymakers and the general public to make informed decisions about EUVR exposure.

## 2 Material and methods

### 2.1 Study area

**Figure 1:**
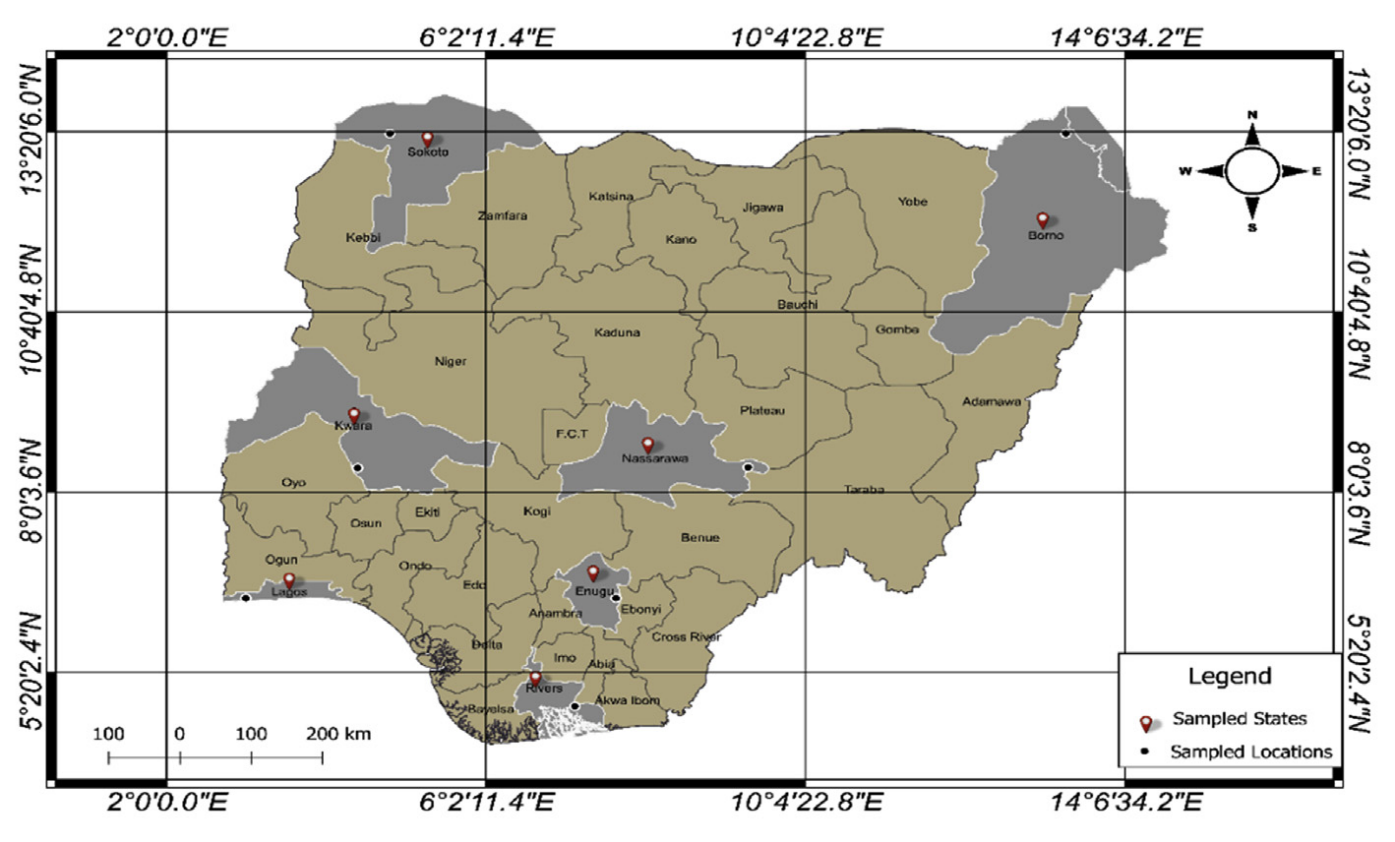
Map of Nigeria showing the study sites marked with black dots.

Nigeria is a West African country with a landmass area of 923,768 km^2^ and a total coastline length of 850 km. It lies between latitudes 4° N - 14° N and longitude 3° E - 15° E (see Figure 1). Nigeria has two major seasons (dry and rainy) and 4 major climate zones. The climatic zones according to Koppen climate classification are the warm desert climate in the northeast, the warm semi-arid climate in the other parts of the north, the monsoon climate in the Niger-Delta, and the tropical savannah climate in the middle belt and parts of the southwest (Akande et al., 2017). The study sites for this research are as follows: 13° N - Borno and Sokoto States, 8° N - Kwara and Nasarawa States, 6° N - Lagos and Enugu States and 4° N - Rivers State which represents the various climate zones.

### 2.2 Data collection and analysis

Satellite data from satellite instruments as shown by Kerr and Fioletov (2008) as one of the resources for studying solar UV radiation was used in this study. Satellite data estimates levels of surface UV radiation using measurements of radiation backscattered to space (atmosphere) with computer models. The data for this study were obtained from the NASA GIOVANNI (GES DISC, Interactive Online Visualization and Analysis Infrastructure), the web interface hosts various satellite instruments used to visualize and analyse geophysical parameters (https://giovanni.gsfc.nasa.gov/giovanni/). Erythemal UV radiation data at noon was extracted from TOMS NIMBUS-7, TOMS EP and OMI satellites from 1979 to 2020. The data obtained were statistically analysed using time series plots, histogram mean plots, ANOVA plots comparing different locations, and box and whisker plots.

## 3 Results and discussion

**Figure 2:**
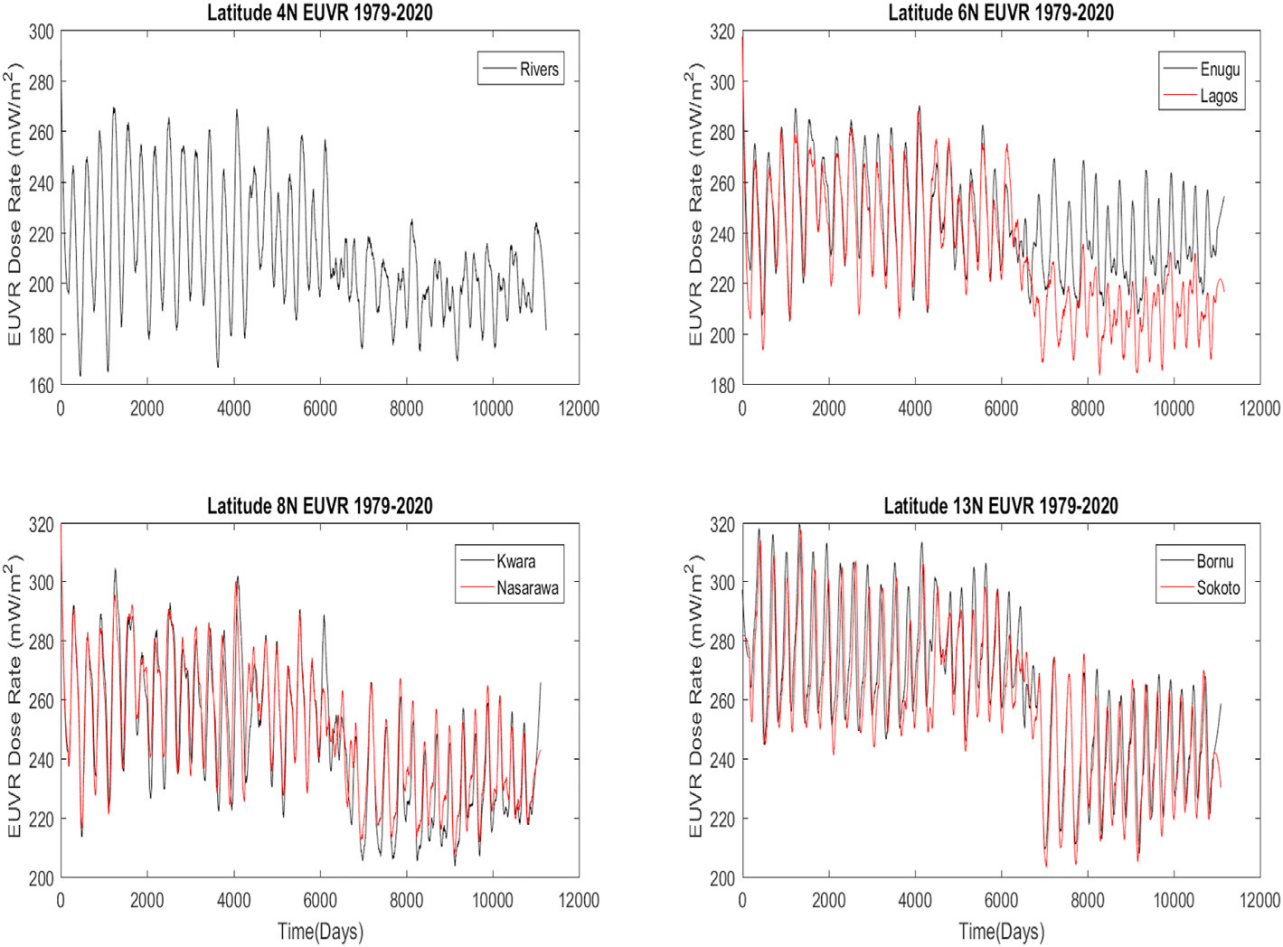
Time series plots of Erythemal UV radiation across various latitudes. Locations on the same latitude are on the same pan.

**Figure 3:**
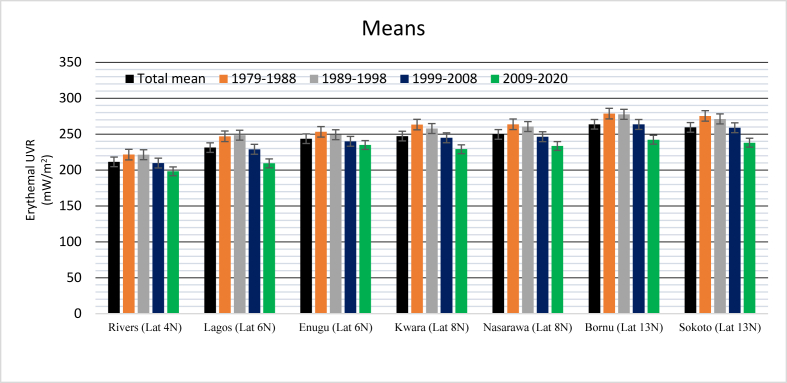
Histogram mean plot of EUVR abundance over the decades.

Figure 2 shows the time series for the four different latitudes studied. The analysis shows that across latitudes, erythemal UV radiation is on the decrease over the entire study period (see also Figure 3). Over the decades as shown in Figure 3, the histogram mean plot of EUVR abundance observed that the values dropped significantly from 1999–2020 compared to the previous years.

**Figure 4:**
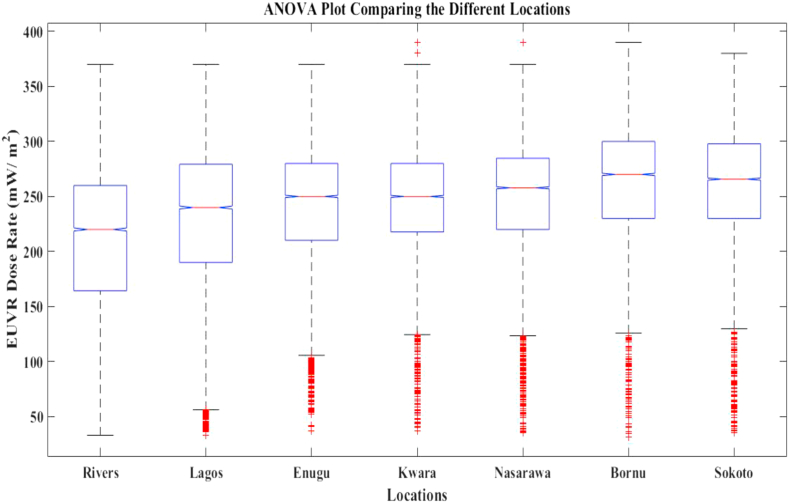
Mean EUVR across latitudes.

Consequently, EUVR varies across all latitudes, the lowest values were recorded in Rivers State (Lat. 4^o^ N), followed by Lat. 6^o^ N (Lagos & Enugu States) then Lat. 8^o^ N (Kwara & Nasarawa States) and lastly Lat. 13^o^ N (Borno & Sokoto States). A critical observation shows that locations higher in latitude recorded higher EUVR values (Figure 4) which is a representation of the different climate zones (from high to low canopy/vegetation cover).

Figure 4 and Table 2, give the mean values of EUVR for the different locations under review. It was observed that the highest mean value was recorded in Borno State at 263.7 (mW/m^2^) followed by Sokoto State at 259.3 (mW/m^2^) while Rivers State recorded the least EUVR at 211.4 (mW/m^2^) (Figure 4).

**Table 2:** Mean values of EUVR for different locations from 1979–2020.

Locations	Total mean	1979–1988	1989–1998	1999–2008	2009–2020
Rivers State (Lat 4^o^ N)	211.4	221.5	221.4	209.7	198.2
Lagos State (Lat 6^o^ N)	231.2	247.0	248.5	228.9	209.3
Enugu State (Lat 6^o^ N)	243.7	253.2	249.2	240.0	234.9
Kwara State (Lat 8^o^ N)	247.3	263.3	257.8	244.9	229.0
Nasarawa State (Lat 8^o^ N)	249.7	263.7	260.6	246.4	233.5
Borno State (Lat 13^o^ N)	263.7	278.6	277.6	263.5	242.2
Sokoto State (Lat 13^o^ N)	259.3	275.4	271.2	258.9	238.1

The figure above (Figure 4) also shows the significant variations in the means of EUVR abundance at various latitudes and locations. When all the locations were compared, the results indicated latitudinal and longitudinal variations. Every location on the eastern axis recorded higher values than its western counterpart on the same latitude. The difference between the total mean value in Enugu (east) & Lagos (west) is 12.5 (mW/m^2^), Nasarawa (east) & Kwara (west) is 2.4 (mW/m^2^) and Borno (east) & Sokoto (west) 6.4 (mW/m^2^) (Table 2). These latitudinal variations can be attributed to the fact that locations in the east receive solar energy before the west. Table 2 is the mean decadal characterization of EUVR abundance, the values recorded showed EUVR decrease across all sites over the years. These values are still far above the WHO standard (see Table 1) dose exposure of 150, all the locations studied had exposures within the high and very high range categories. This implies that Nigerians have been receiving an overdose of EUVR for a very long time now which has a lot of health implications such as skin cancer etc. The result of this study is in line with the data from Weather Online (2021) which shows that most African countries have a very high UV index.

**Figure 5:**
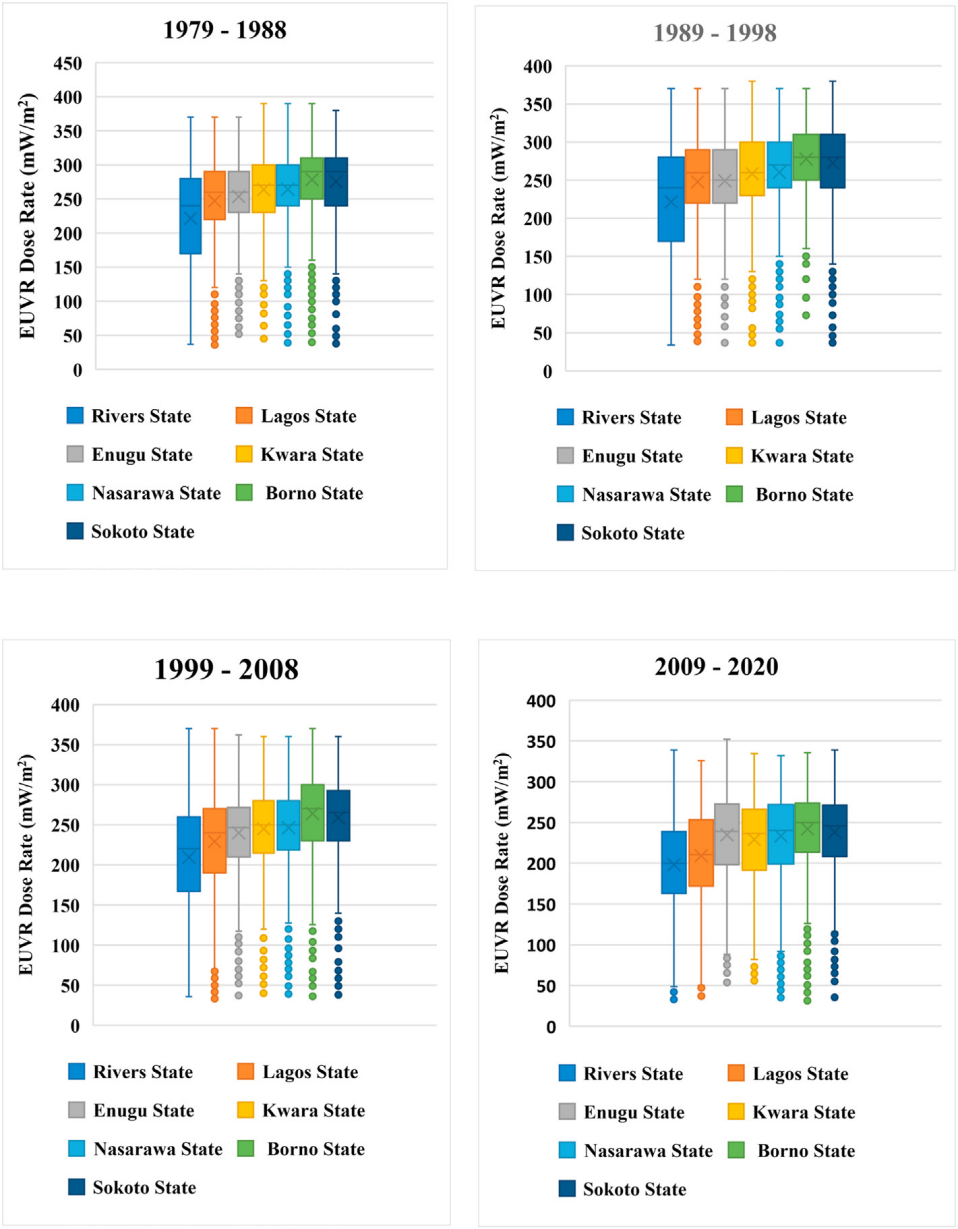
5_a_–5_d_: Box and Whisker Plots for Mean Multi-year EUVR across various latitudes and phases.

Furthermore, Box and whisker plots were used to summarize the latitudinal variance in the mean multi-year EUVR at each phase of this study (see Figure 5). The computed results consist of the median, interquartile (Q1), interquartile (Q3) and mean values. The results obtained across various phases were shown in Figure 5*a*–*d*.

The results from Figure 5*d* (2009–2020) were very significant because they recorded the phase with minimum mean multi-year EUVR across all the latitudes studied. The maximum and minimum whiskers from 2009 – 2020 were 351.9 (mW/m^2^) and 31.3 (mW/m^2^) respectively, and they are lower than the mean multi-year EUVR from previous phases i.e., 1979–1988, 1989–1998 and 1999–2008. From the maximum whiskers above, it was observed that the 1979–1988 phase had the highest mean multi-year EUVR which can be attributed to the period of high emission of ozone-depleting substances (ODS) before the implementation of the Montreal Protocol in 1989 which rescued humans from extreme exposure to solar UV radiation. The notable downward trend in mean multi-year EUVR from 1979–2020 can be attributed to United Nations programs to ensure the healing of the ozone layer across the world. Over the years, the Nigerian government have instituted regulations to monitor the environmental impact of her crude oil explorations and the reduction in mean multi-year EUVR from 2009–2020 is one of the positive impacts of such policies.

## 4 Conclusion

The harmful effects of solar ultraviolet (UV) radiation on human beings remain a public health concern that needs to be investigated. Furthermore, knowledge of EUVR distribution across various latitudes in Nigeria is essential because of its public health relevance given that many Nigerians are ignorant of the health detriments of high exposure to EUVR. It was therefore the purpose of this study to analyse the latitudinal variations in mean daily and multi-year erythemal ultraviolet radiation (EUVR) across various climate belts in Nigeria. It was found that the mean daily EUVR at the selected latitudes ranged from 32.9 to 390 mW/m^2^. The results also showed that Borno State located at latitude 13° N had the highest EUVR, while Rivers State recorded the least EUVR at latitude 4° N. Also, longitudinal differences play a vital role in the level of EUVR exposure, while eastern longitudes receive more erythemal exposures. This study is a guide to understanding the UV index across various latitudes in Nigeria. It will also serve as a reference for future scientific research on the variation of EUVR over Nigeria. Finally, this study has shown that EUVR exposure across Nigeria is above the recommended dosage for humans.

## 5 Recommendations

This study recommends the suggestions of the WHO (2002) in preventing the effects of solar UV radiation which include: (i). Limiting long exposure of the skin to solar UV, especially during midday. (ii). Staying in overhead sheds and wearing skin protective coverings habitually. (iii). Use of sunshades to protect the eyes. (iv). Use and reapplication of broad-spectrum sunscreen products with SPF 15+ (Sun Protecting Factor) on the skin regularly. iv. Avoiding Tanning beds. (v). The Nigerian government should through its public awareness programs educate and heighten the awareness of the deleterious effects of EUVR.

## Declarations

### Author contribution statement

Timothy C. Egbuim; Nnaemeka D. Onyeuwaoma: Conceived and designed the experiments; Performed the experiments; Analyzed and interpreted the data; Wrote the paper.

Bonaventure I. Okere: Conceived and designed the experiments; Performed the experiments; Analyzed and interpreted the data.

Mercy H. Ezenwugo; Augustina O. Chukwudi; Godspower O. Uhiene; Ngozi D. Ugwuozor; Baba I. Shaibu: Conceived and designed the experiments; Performed the experiments; Contributed reagents, materials, analysis tools or data.

Emeka A. Ugboma; Daniel R.E. Ewim: Analyzed and interpreted the data.

### Funding statement

This research did not receive any specific grant from funding agencies in the public, commercial, or not-for-profit sectors.

### Data availability statement

The data for the study was generated from NASA GIOVANNI online data web for atmospheric sciences.

### Declaration of interest's statement

The authors declare no conflict of interest.

### Additional information

No additional information is available for this paper.
